# Central precocious puberty in Türkiye, 2018–2024: national incidence, prevalence, and changes across the COVID-19 period

**DOI:** 10.3389/fendo.2026.1773426

**Published:** 2026-03-10

**Authors:** Emre Özer, Seçil Çakır Gündoğan, Abdurrahman Bitkay, Neslihan Öztürk, Naim Ata, Şuayip Birinci, Aylin Kılınç Uğurlu, Onur Akın

**Affiliations:** 1 Department of Pediatric Endocrinology, Gülhane Training and Research Hospital, Ankara, Türkiye; 2 Department of Pediatric Endocrinology, Etlik City Hospital, Ankara, Türkiye; 3 Department of Pediatric Endocrinology, Yenimahalle Training and Research Hospital, Ankara, Türkiye; 4 Department of Health Information Systems, Republic of Türkiye Ministry of Health, Ankara, Türkiye; 5 Department of Strategy Development, Republic of Türkiye Ministry of Health, Ankara, Türkiye; 6 Deputy Minister of Health, Republic of Türkiye, Ankara, Türkiye; 7 Department of Pediatric Endocrinology, Faculty of Medicine, Gazi University, Ankara, Türkiye; 8 Department of Pediatric Endocrinology, Gülhane Faculty of Medicine, University of Health Sciences, Ankara, Türkiye

**Keywords:** central precocious puberty (CPP), COVID - 19, electronic health records - EHR, incidence, prevalence, türkiye

## Abstract

**Background:**

Rates of central precocious puberty (CPP) are increasing worldwide; however, contemporary population-based studies remain limited in many settings, including Türkiye, and temporal patterns across the COVID-19 period are incompletely characterised. To address these gaps we aimed to quantify national incidence and prevalence of treated CPP from 2018 to 2024 and to describe temporal patterns across the COVID-19 period and geographic variation.

**Methods:**

We conducted a retrospective, population-based registry study using Türkiye’s national electronic health record system (e-Nabız). For incidence analyses, we included girls aged <10 years and boys aged <11 years between Jan.1,2018, and Dec.31,2024; prevalence analyses included girls aged ≤11 years and boys aged ≤12 years to capture ongoing treatment. CPP cases were identified using a combined diagnostic and treatment algorithm requiring pediatric endocrinology evaluation, CPP-related ICD-10 coding at point of care, and sustained GnRHa treatment (≥3 prescriptions issued on separate dates, with a minimum interval of 15 days). Annual incidence and prevalence were calculated using Turkish Statistical Institute denominators and stratified by sex, year, and region.

**Results:**

We identified 41–169 treated CPP cases, 95.6% in girls (39380/41169). National incidence increased from 25 to 54 per 100,000 between 2018 and 2024, and prevalence from 23 to 112 per 100 000. Annual case counts peaked following the COVID-19 period (3816 in 2019 to 7653 in 2021) and subsequently plateaued above pre-pandemic levels.

**Conclusion:**

In this national, population-based study from Türkiye, the incidence of treatment-initiated CPP increased substantially between 2018 and 2024, with a pronounced rise during the COVID-19 period and persistently higher levels thereafter. These findings are relevant for pediatric endocrine service planning and population-level public health strategies, but should be interpreted as temporal trends in specialist-confirmed, treatment-requiring CPP rather than the full clinical spectrum of CPP (including the pandemic period) or a direct measure of biological pubertal onset in the general population.

## Introduction

Precocious puberty (PP) is defined by the development of secondary sexual characteristics before the age of 8 years in girls and 9 years in boys. The most common form is central precocious puberty (CPP), caused by premature activation of the hypothalamic–pituitary–gonadal (HPG) axis, although peripheral mechanisms may underlie other forms of PP (1). PP can have clinically important consequences, including compromised adult height and psychosocial effects, and has been associated with adverse long-term health outcomes, including cardiometabolic diseases and certain cancers (2). Over recent decades, multiple populations have reported a secular trend toward earlier pubertal timing. Proposed contributors include changes in nutrition and adiposity, as well as exposure to endocrine-disrupting chemicals (EDC); however, the relative contributions and underlying mechanisms remain incompletely understood (3–6). Population-based estimates of CPP vary widely across settings, reflecting differences in diagnostic thresholds, ascertainment strategies, healthcare access, and environmental context.

Population-based studies have documented substantial increases in CPP in several countries, although reported rates vary widely. For example, national registry data from Denmark (1998–2017) showed a marked rise in CPP incidence, and population-based analyses from the Republic of Korea also reported steep increases over time (7, 8). Other reports from Europe and Asia further demonstrate pronounced between country variability, likely reflecting differences in diagnostic thresholds, ascertainment strategies, healthcare access and environmental context (9–11). Collectively, these findings underscore geographic heterogeneity and motivate contemporary, population-level estimates across diverse health systems.

The COVID-19 pandemic created a time delimited, population level natural experiment, with marked changes in children’s routines including reduced physical activity, increased sedentary behaviour and screen exposure, and weight gain that may influence pubertal timing. However, existing evidence is derived largely from clinic based cohorts or limited administrative samples, which may not reflect the national burden and often cannot characterise temporal patterns at scale. To address these gaps, we used Türkiye’s national electronic health record (EHR) system to quantify national incidence and prevalence of CPP requiring treatment from 2018 to 2024 and to describe temporal patterns across the COVID-19 period and geographic variation.

## Subjects and methods

We conducted a retrospective, nationwide, population-based registry study using data from Türkiye’s national EHR system, e-Nabız, to examine treated CPP during Jan. 1, 2018, to Dec. 31, 2024. Administered by the Ministry of Health, e-Nabız integrates routinely collected data from public and private healthcare providers nationwide, including demographics, International Classification of Diseases, 10th Revision (ICD-10) diagnoses, prescription records and selected clinical data. The platform is linked to the Social Security Institution’s Medulla reimbursement system, supporting near-complete capture of treated cases and healthcare utilisation across Türkiye. This population-level coverage enabled national estimates of treated CPP incidence and prevalence and assessment of temporal patterns across the COVID-19 period and geographic variation.

### Study population and case definition

The study population comprised children in Türkiye who presented to public or private healthcare facilities between January 1, 2018, and December 31, 2024. In routine pediatric endocrinology practice, CPP is characterized by (i) onset of secondary sexual characteristics before age 8 years in girls and before age 9 years in boys, (ii) pubertal progression on serial examinations (Tanner staging), (iii) advanced bone age relative to chronological age, and (iv) biochemical evidence of HPG axis activation, typically assessed by basal and/or GnRH stimulation testing (pubertal LH response), with estradiol/testosterone as appropriate; and pelvic ultrasonography in girls. Because these clinical, hormonal, and imaging details are not fully available at the individual level in the e-Nabız database, case ascertainment used a combined diagnostic-and-treatment algorithm requiring: (i) evaluation in pediatric endocrinology clinics, (ii) recording of CPP-related ICD-10 diagnostic codes at the point of care, and (iii) GnRH agonist prescriptions (ATC L02AE02, L02AE04). Analyses were restricted to central CPP treated with GnRH agonists. To increase specificity for sustained, treatment-requiring disease and reduce misclassification from isolated, erroneous, or trial prescriptions, CPP was operationally defined as ≥3 GnRH agonist prescriptions issued on separate dates at least 15 days apart. This threshold aligns with the national reporting/reimbursement framework and was therefore used as a pragmatic marker of treatment continuity. This case-ascertainment strategy provides a reproducible operational approximation of treatment-requiring CPP at the population level in a nationwide administrative dataset lacking individual-level clinical adjudication variables. Similar coding-plus-treatment frameworks have been used in national registry/claims-based CPP epidemiology (7, 8, 10). For incidence analyses, cases were included if treatment initiation occurred before age 10 years in girls and before age 11 years in boys. For prevalence analyses, we included girls aged ≤11 years and boys aged ≤12 years to capture all children receiving ongoing therapy during the study period.

### Data cleaning

Data cleaning procedures included removal of duplicate records based on unique patient identifiers, exclusion of conflicting entries (such as duplicate prescriptions recorded on the same day), and exclusion of records with missing key variables. Children with duplicate identifiers or fewer than three qualifying GnRH agonist prescriptions were excluded from all analyses.

### Measurement parameters

Annual incidence was defined as the number of newly identified cases of central precocious puberty per calendar year divided by the corresponding mid-year child population (girls aged ≤10 years; boys aged ≤11 years), using official mid-year population counts from the Turkish Statistical Institute (TÜİK). Annual prevalence was defined as the number of children receiving active treatment for central precocious puberty in a given year divided by the corresponding child population (girls aged ≤11 years; boys aged ≤12 years). Residential location was obtained from the Ministry of Health Family Medicine Unit registry and categorised by province and geographic region.

### Statistical analysis

Statistical analyses were conducted using SPSS version 25.0 (IBM Corp., Armonk, NY, USA). Continuous variables were summarised as mean (standard deviation) or median (minimum–maximum), as appropriate, and categorical variables as counts and percentages.

## Results

Sex and age distribution: From 2018 to 2024, we identified 41–169 treated CPP cases; 39 380 (95.6%) were girls and 1 789 (4.4%) were boys. The proportion of boys was similar across years (3.4%–5.6%). Detailed annual distributions of treated CPP cases by sex and single-year age groups are provided in **Table 1**.

**Table 1:** Year, sex and age distribution.

Years	Sex	0 Age	1 Age	2 Age	3 Age	4 Age	5 Age	6 Age	7 Age	8 Age	9 Age	10 Age	11 Age	Total
2018	Boys			1	1	2	5	5	11	29	63	54	30	201
	Girls		2	4	12	18	29	88	296	999	1367	627		3442
2019	Boys		1		2	5	5	7	15	32	52	64	30	213
	Girls		2	5	10	15	25	86	323	977	1446	714		3603
2020	Boys			1	1		2	6	11	21	61	41	12	156
	Girls	1	2	4	8	13	47	146	596	1420	1230	413		3880
2021	Boys			1	7	5	10	5	12	33	85	77	28	263
	Girls		3	3	7	10	45	171	736	2404	3043	968		7390
2022	Boys					4	8	12	21	54	92	112	46	349
	Girls		4	5	8	18	68	149	522	1874	3040	1535		7223
2023	Boys			1	1		7	12	16	45	92	83	39	296
	Girls			5	6	12	40	127	527	1949	2880	1384		6930
2024	Boys				1	3	3	10	13	48	79	104	50	311
	Girls			6	4	8	40	129	481	1793	2913	1538		6912
Total	Boys		1	4	13	19	40	57	99	262	524	535	235	1789 (4.35%)
	Girls	1	13	32	55	94	294	896	3481	11416	15919	7179		39380 (95.65%)
Total		1	14	36	68	113	334	953	3580	11678	16443	7714	235	41169

### Incidence

Overall national incidence of treated CPP increased from 25 to 54 per 100–000 children between 2018 and 2024. When stratified by sex, incidence rose from 50 to 111 per 100–000 among girls and from 2 to 5 per 100–000 among boys over the study period. Incidence nearly doubled between 2020 (28 per 100,000) and 2021 (53 per 100,000) and then remained elevated through 2024 (52–54 per 100,000), indicating a pandemic-era rise followed by a plateau above pre-pandemic levels. Annual incidence rates by sex, together with region are provided in **Table 2**.

**Table 2:** Incidence by region and sex (per 100,000).

Regions	Sex	2018	2019	2020	2021	2022	2023	2024
Mediterranean Region	Boys	2	2	2	3	4	4	3
	Girls	47	46	45	80	98	87	94
	Total	23	23	22	38	48	42	45
Eastern Anatolia Region	Boys	1	1	0	1	2	2	2
	Girls	22	21	25	32	51	50	63
	Total	11	10	12	15	25	24	31
Aegean Region	Boys	2	2	2	5	5	5	6
	Girls	46	50	57	118	98	96	87
	Total	22	24	28	57	48	47	44
Southeastern Anatolia Region	Boys	1	1	1	1	2	1	2
	Girls	24	32	41	56	61	60	71
	Total	12	16	20	27	30	29	34
Central Anatolia Region	Boys	5	4	3	6	8	7	8
	Girls	96	101	105	206	195	208	203
	Total	47	49	51	99	95	100	98
Black Sea Region	Boys	3	4	3	6	7	7	5
	Girls	48	55	47	118	117	122	126
	Total	24	27	23	58	58	60	61
Marmara Region	Boys	3	4	2	3	5	4	4
	Girls	53	52	61	125	117	111	114
	Total	26	26	29	60	57	54	55
Total	Boys	3	3	2	3	5	4	4
	Girls	50	53	57	110	109	108	111
	Total	25	26	28	53	53	52	54

Incidence was calculated per 100,000 population using individuals aged 11 and under for boys, and 10 and under for girls.

### Prevalence

Overall prevalence of treated CPP increased from 23 per 100–000 children in 2018 to 112 per 100–000 in 2024. Prevalence increased from 46 to 230 per 100–000 among girls and from 2 to 10 per 100–000 among boys. The steepest expansion occurred during and immediately after the pandemic onset (overall: 23 in 2018 → 51 in 2020 → 108 in 2022), with prevalence remaining high in 2023–2024 (113–112 per 100,000). Annual prevalence rates by sex, together with region- and province-specific estimates, are provided in **Table 3**.

**Table 3:** Prevalence by region and sex (per 100,000).

Regions	Sex	2018	2019	2020	2021	2022	2023	2024
Mediterranean Region	Boys	2	4	4	5	7	9	9
	Girls	43	76	89	115	183	194	195
	Total	21	37	44	56	89	95	95
Eastern Anatolia Region	Boys	1	1	1	1	3	4	5
	Girls	21	36	46	52	91	106	121
	Total	10	17	22	25	44	52	59
Aegean Region	Boys	2	3	4	7	11	12	14
	Girls	42	79	105	163	218	218	202
	Total	21	38	51	80	107	108	101
Southeastern Anatolia Region	Boys	1	2	2	2	4	4	4
	Girls	22	49	74	91	129	136	147
	Total	11	24	36	44	62	66	71
Central Anatolia Region	Boys	4	7	7	9	14	16	17
	Girls	87	159	186	277	386	397	391
	Total	43	78	91	134	187	194	191
Black Sea Region	Boys	2	6	7	10	15	17	16
	Girls	44	86	95	157	240	262	272
	Total	22	43	48	78	119	131	135
Marmara Region	Boys	3	6	6	5	9	10	10
	Girls	49	87	108	170	246	253	244
	Total	24	44	54	82	120	123	118
Total	Boys	2	4	5	5	9	10	10
	Girls	46	84	105	153	222	231	230
	Total	23	42	51	74	108	113	112

Prevalence was calculated per 100,000 population using individuals aged 12 and under for boys, and 11 and under for girls.

### Pandemic-period temporal pattern

**Figure 1:**
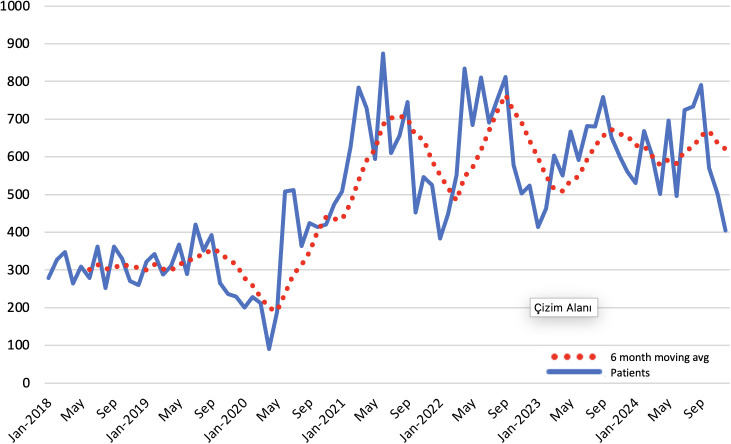
Monthly number of patients newly initiated on GnRHa treatment and 6-month moving average.

Age distribution analyses suggested a pandemic-period shift in treated case composition. Among girls, the proportion diagnosed at age 8 increased from 27.1% (2019) to 36.6% (2020), while age 10 decreased from 19.8% to 10.6%. Among boys, age 9 increased from 24.4% (2019) to 39.1% (2020). These patterns during the pandemic period are presented in **Table 4**. In additional temporal analyses (quarterly visualization), case counts declined during the early pandemic disruption phase (mid-2020) and then rebounded, peaking around mid-2021. Thereafter, counts remained above pre-pandemic levels through 2023–2024 (**Figure 1**).

**Table 4:** Annual sex-specific age distribution of treated central precocious puberty.

Year	Sex	<=6 y.o.	7 y.o.	8 y.o.	9 y.o.	10 y.o.	11 y.o.	Total
2018	Female	4,4%	8,6%	29,0%	39,7%	18,2%		3442
2019	Female	4,0%	9,0%	27,1%	40,1%	19,8%		3603
2020	Female	5,7%	15,4%	36,6%	31,7%	10,6%		3880
2021	Female	3,2%	10,0%	32,5%	41,2%	13,1%		7390
2022	Female	3,5%	7,2%	25,9%	42,1%	21,3%		7223
2023	Female	2,7%	7,6%	28,1%	41,6%	20,0%		6930
2024	Female	2,7%	7,0%	25,9%	42,1%	22,3%		6912
2018	Male	7,0%	5,5%	14,4%	31,3%	26,9%	14,9%	201
2019	Male	9,4%	7,0%	15,0%	24,4%	30,0%	14,1%	213
2020	Male	6,4%	7,1%	13,5%	39,1%	26,3%	7,7%	156
2021	Male	10,6%	4,6%	12,5%	32,3%	29,3%	10,6%	263
2022	Male	6,9%	6,0%	15,5%	26,4%	32,1%	13,2%	349
2023	Male	7,1%	5,4%	15,2%	31,1%	28,0%	13,2%	296
2024	Male	5,5%	4,2%	15,4%	25,4%	33,4%	16,1%	311

### Geographic variation

CPP rates varied across Türkiye. Central Anatolia region consistently had the highest incidence, whereas Eastern Anatolia region had the lowest, in both sexes. Province and region specific estimates are provided in **Table 2** and **3** and in **Supplementary Table 1** and **2**.

## Discussion

This nationwide registry study shows that therapy initiated CPP increased in Türkiye between 2018 and 2024, with the largest rise following the COVID-19 period and levels remaining higher than before the pandemic. Using linked national EHR data, we provide population level estimates from a large country in the WHO European Region with broad paediatric healthcare coverage, reducing selection bias seen in single-centre or subnational studies.

Girls accounted for the majority of treated cases in our cohort, consistent with population based studies from other settings reporting a similar female predominance. In national registry or population based studies from Denmark, the Republic of Korea, and France, girls have consistently comprised about 90%–95% of treated or registered CPP cases (7, 8, 10, 12, 13). The comparable sex distribution in our study suggests that female predominance in CPP persists across countries and likely reflects greater underlying biological predisposition in girls.

### Global comparison of incidence and prevalence

CPP incidence in Türkiye was comparable to several European estimates but remained substantially lower than reports from East Asia. Among girls, incidence in Türkiye reached 111 per 100–000 in 2024. This is close to the mean annual incidence reported from Denmark (≈92 per 100–000 girls, 1998–2017) and higher than the incidence reported from France for girls younger than 9 years (26.8 per 100 000), while remaining ~6-fold lower than Taiwan (≈702 per 100–000 girls <10 years) and ~13-fold lower than the Republic of Korea (≈1–415 per 100–000 girls in 2020). Among boys, Türkiye’s incidence in 2024 was 4 per 100 000, compared with ≈9 per 100,000 (mean annual) in Denmark, 2.4 per 100–000 in France, and much higher rates reported in Taiwan (57.2 per 100 000) and Korea (≈100 per 100 000) (7, 8, 10–12).

CPP prevalence in Türkiye also increased over the study period. In 2024, prevalence reached 230 per 100–000 among girls and 10 per 100–000 among boys. This is similar in level to early Danish prevalence outcomes (≈200 per 100–000 girls and <50 per 100–000 boys in birth cohorts from 1992–1993), while remaining substantially lower than estimates reported from East Asia, including Korea (≈657 per 100–000 girls and ≈28 per 100–000 boys in 2020; overall ≈319 per 100 000) and Taiwan (≈1–109 per 100–000 girls and ≈70 per 100–000 boys over 2000–2013) (8, 11, 13). Methodological heterogeneity is a major contributor to cross-country differences in reported CPP incidence and prevalence. In nationwide claims- and registry-based epidemiology, detailed individual-level clinical variables are often unavailable; therefore, case ascertainment commonly relies on diagnostic coding linked to claims, prescription, and reimbursement data. Accordingly, several national studies including those from Korea (Kang et al., 2023), Denmark (Braüner et al., 2020), and France (Le Moal et al., 2018) have used comparable coding-plus-treatment ascertainment strategies based on diagnostic coding and linked claims/prescription/reimbursement data (7, 8, 10). In this context, outcomes are most appropriately interpreted as treatment-requiring (treated) CPP rather than the full clinical spectrum of CPP. In addition, differences in case definitions can substantially influence reported incidence and prevalence. Broader case definitions especially those emphasising early physical signs and advanced bone age may capture benign pubertal variants (e.g., premature thelarche or adrenarche), inflating prevalence estimates. Variation in screening intensity, access to specialist care, and environmental exposures (including EDCs) may also contribute. The potential impact of misclassification is illustrated in Danish registry research, where a clinical review of 100 registry-identified precocious puberty cases confirmed CPP in 46% of children, while the remainder were benign variants (9, 13–17). Although the 2022 Korean Clinical Practice Guidelines for CPP are broadly aligned with practice in Türkiye, higher estimates reported from Korea may partly reflect differences in detection pathways and access to specialised care (18). Similarly, higher estimates from Taiwan may partly reflect diagnostic approaches that combine early physical maturation with advanced bone age criteria. Accordingly, cross-country comparisons should be interpreted cautiously because age thresholds, case definitions (treated vs. diagnosed), and ascertainment and coding practices differ across settings. Nevertheless, nationwide e-Nabız coverage with reimbursement linkage provides more generalisable population-level estimates of treated CPP than single-centre or subnational datasets (9, 14–17). In addition, inclusion of pre-, peri-, and post-pandemic periods within the same national data infrastructure enables a clearer assessment of temporal trends, with direct relevance to paediatric health-service planning.

### Trends in precocious puberty during the COVID-19 pandemic

Evidence linking the COVID-19 period to increases in CPP has largely come from single centre or multicentre, hospital-based series. Using Türkiye’s EHR, we provide population-based estimates of treated CPP across the pre-pandemic, pandemic, and post-pandemic periods. Annual case counts increased from 3,816 in 2019 to a peak of 7,653 in 2021, then declined modestly to 7,223 in 2024, remaining above pre-pandemic levels.

The pandemic-period analyses suggest a three-phase temporal pattern in treated CPP: an early decline during the disruption phase, a rebound with a peak around mid-2021, and persistently elevated case counts thereafter compared with pre-pandemic years. Given the registry-based observational design, this pattern is best interpreted as a temporal association, while causal attribution remains beyond the scope of the present analysis. Several non-mutually exclusive mechanisms may have contributed, including shifts in healthcare-seeking behavior and referral pathways, changes in diagnostic intensity, and delayed presentation followed by catch-up evaluation and treatment after restrictions eased. Notably, the persistence of elevated levels through 2023–2024 suggests that deferred care catch-up alone may not fully account for the post-pandemic pattern. Age-distribution findings also indicate a change in the composition of treated cases during the pandemic period. In girls, the relative proportion at age 8 increased while age 10 decreased in 2020 versus 2019; in boys, the proportion at age 9 increased over the same interval. These compositional shifts are compatible with changes in the timing of specialist presentation and treatment initiation during the pandemic, rather than a uniform population-wide shift in biological pubertal onset alone. In addition, clinician- and center-level variation in the threshold to initiate GnRHa treatment during the pandemic (e.g., perceived progression, psychosocial concerns, and service capacity constraints) may have influenced treatment initiation and thus the observed registry-based trends. Accordingly, the observed pattern likely reflects, at least in part, changes in detection and treatment practices, although a biological contribution cannot be excluded. The broader international literature also reports increases during the pandemic period, although most studies are clinic-based and therefore more sensitive to referral patterns. In China, the proportion of new-onset precocious puberty among girls increased from 0.4% (2018) to 6.2% (2020) when assessed relative to outpatient visits (19); in a tertiary-centre analysis from the United States, the proportion initiated on GnRH agonist therapy increased from 1.2% pre-pandemic to 2.8% during the pandemic (20); and in Italy, suspected precocious/early puberty accounted for 6.2% of visits in 2019 and 27.0% in 2020, with progressive CPP among evaluated girls increasing from 23.1% to 42.6% (21). Studies from Türkiye have similarly reported more frequent CPP diagnoses and younger pubertal timing during the pandemic (22, 23).

Beyond temporal increases in case counts, several studies have described concurrent shifts in clinical, metabolic, and biochemical profiles during the pandemic period. In Thailand, a single-centre study reported both increased CPP diagnoses and a higher prevalence of overweight/obesity among affected children (30% in 2019 to 46% in 2020), with higher BMI z-scores after pandemic onset, supporting a potential contribution of pandemic-associated weight gain (24). A multicentre study from Türkiye similarly reported earlier pubertal onset during the pandemic, alongside greater initiation of pubertal suppression therapy at presentation (25). Biochemical differences have also been reported: in Shanghai, Chen and colleagues observed higher peak luteinising hormone to follicle-stimulating hormone ratios, higher mean GnRH concentrations, lower sex hormone binding globulin levels, and reduced MKRN3 concentrations in children diagnosed during the pandemic compared with pre-pandemic cohorts; ghrelin and MKRN3 levels were positively correlated among girls with CPP (26).

Taken together, prior reports suggest a consistent international pattern of increased CPP during the COVID-19 period, often accompanied by shifts in clinical, metabolic, or biochemical profiles. These temporal patterns coincided with prolonged lockdowns and marked population-wide lifestyle changes, including reduced physical activity, increased sedentary time and screen exposure, weight gain, and psychosocial stress. These disruptions have been hypothesised to affect neuroendocrine signaling and thereby facilitate earlier activation of the HPG axis (27). However, because many prior studies rely on clinic-based denominators and are sensitive to changes in healthcare-seeking and referral pathways, our linked national EHR and treatment reimbursement design supports robust population-level inference on temporal shifts in treated CPP and, to our knowledge, provides the first nationwide evidence on changes occurring across the COVID-19 lockdown/restriction period findings directly relevant to paediatric health planning.

### Regional variations

Regional variation in treated CPP incidence was evident across Türkiye. Central Anatolia showed consistently higher rates, with Ankara recording the highest province level incidence nationally. Similar geographic clustering has been described in parts of Europe, including the Viareggio area in Italy (36.3 vs. 3.3–10.7 per 100–000 in other regions) and regions of southwestern and central-eastern France, where contextual environmental and sociodemographic factors have been discussed as potential contributors. In those settings, hypotheses have included agricultural and industrial exposures, and EDCs have been proposed as one possible pathway (10, 11, 15). In Türkiye where agricultural activity is widespread urban/rural differences in environmental context may contribute to regional disparities; however, variation in health-system factors is also likely to be important. Ankara’s high rates may partly reflect greater access to specialist care and higher diagnostic intensity, given its concentration of tertiary centres and paediatric endocrinology capacity, which can increase case detection and treatment initiation. Conversely, provinces with limited specialist access may show lower recorded rates; for example, Ardahan, a sparsely populated province, reported no treated CPP cases among girls in 2019 and 2023, which may indicate under-ascertainment rather than true absence. Similar interpretive challenges have been noted in Korea, where rising CPP incidence has been attributed not only to environmental factors but also to heightened parental concern, vigorous marketing and improved access to healthcare (8). Together, these findings suggest that observed geographic differences likely reflect a mix of underlying risk-factor distribution and variation in ascertainment, underscoring the need to interpret regional comparisons cautiously and to align service capacity and surveillance with areas of highest recorded burden.

This study has several limitations. First, the definitive diagnosis of CPP requires pediatric endocrinology specialist assessment, including a detailed history and physical examination, pubertal staging, bone age evaluation, hormonal testing (basal and/or stimulated gonadotropins with estradiol as appropriate), and longitudinal follow-up elements that are not fully captured in routinely collected electronic records. We acknowledge that the absence of individual level clinical and laboratory variables (including pubertal progression, bone age advancement, and gonadotropin profiles) precludes direct confirmation of each diagnosis. Therefore, our findings should be interpreted as national trends in specialist-confirmed, therapy-initiated (treatment-requiring) CPP, rather than as a direct measure of biological pubertal onset in the general population. Second, we could not capture children with CPP who were not treated with GnRH agonists, and some degree of misclassification may persist despite our stringent treatment-based algorithm. Third, our analyses are primarily descriptive; although age-restricted denominators mitigate age-structure effects, we did not apply formal age standardization or regression-based trend modeling, which may limit comparability of crude rates over time. Fourth, because data were available from 2018 onward, we were unable to evaluate longer-term secular trends before the study period.

This study also has important strengths. Using a nationwide electronic health record system with near complete coverage of paediatric healthcare utilisation in Türkiye (and linkage to reimbursement data) reduces the selection bias inherent in single-centre or subnational reports and enables population-level estimation. Our strict case definition was designed to improve specificity for treated CPP, and the inclusion of pre-, peri- and post-pandemic years within a single national framework strengthens inference regarding temporal changes. Collectively, these features provide strong national estimates of treated CPP and offer evidence relevant to paediatric endocrine service planning and broader child health policy and prevention priorities.

## Conclusion

In this nationwide, population-based registry study using linked electronic health records data, we observed a substantial increase in therapy-initiated (treated), CPP in Türkiye from 2018 to 2024, with a pronounced rise following the COVID-19 period and persistently elevated levels thereafter. Although the etiology of CPP remains incompletely understood, the pandemic-associated increase underscores the importance of supporting healthy daily routines in children during prolonged societal disruptions. Temporal and geographic variation further suggests aligning pediatric endocrine capacity and referral pathways with higher-burden areas and strengthening registry surveillance using harmonized, transparent case definitions.

## Data Availability

The original contributions presented in the study are included in the article/**Supplementary Material**. Further inquiries can be directed to the corresponding author.
