# Supplementary material for: Central precocious puberty in Türkiye, 2018–2024: national incidence, prevalence, and changes across the COVID-19 period
Source: Front Endocrinol (Lausanne). 2026 Mar 10;17:1773426. doi: 10.3389/fendo.2026.1773426 (PMC13008741; doi:10.3389/fendo.2026.1773426)
Supplement: Supplementary file 1 [file Table1.docx]

Supplementary Table 1. Incidence by Province and Sex (per 100,000 People)

|  |  | 2018 | 2019 | 2020 | 2021 | 2022 | 2023 | 2024 |  |  | 2018 | 2019 | 2020 | 2021 | 2022 | 2023 | 2024 |
| --- | --- | --- | --- | --- | --- | --- | --- | --- | --- | --- | --- | --- | --- | --- | --- | --- | --- |
| ADANA | Boys | 1 | 1 | 3 | 3 | 5 | 2 | 1 | KAHRAMANMARAŞ | Boys | 3 | 2 | 1 | 2 | 2 | 0 | 1 |
|  | Girls | 36 | 41 | 46 | 79 | 74 | 70 | 72 |  | Girls | 48 | 52 | 52 | 75 | 84 | 60 | 82 |
| ADIYAMAN | Boys | 3 | 0 | 0 | 0 | 1 | 0 | 0 | KARABÜK | Boys | 0 | 6 | 0 | 6 | 13 | 13 | 7 |
|  | Girls | 30 | 12 | 15 | 33 | 71 | 43 | 65 |  | Girls | 105 | 114 | 87 | 155 | 143 | 137 | 173 |
| AFYONKARAHİSAR | Boys | 0 | 0 | 2 | 3 | 3 | 3 | 13 | KARAMAN | Boys | 0 | 4 | 4 | 0 | 0 | 0 | 0 |
|  | Girls | 33 | 53 | 57 | 137 | 92 | 99 | 125 |  | Girls | 5 | 15 | 10 | 51 | 78 | 90 | 71 |
| AĞRI | Boys | 0 | 0 | 0 | 1 | 3 | 2 | 0 | KARS | Boys | 0 | 0 | 0 | 0 | 4 | 0 | 8 |
|  | Girls | 4 | 4 | 6 | 17 | 26 | 26 | 20 |  | Girls | 14 | 7 | 11 | 24 | 46 | 22 | 72 |
| AKSARAY | Boys | 2 | 2 | 0 | 2 | 7 | 0 | 0 | KASTAMONU | Boys | 0 | 0 | 0 | 17 | 13 | 9 | 9 |
|  | Girls | 51 | 52 | 63 | 181 | 139 | 160 | 131 |  | Girls | 83 | 95 | 58 | 148 | 115 | 156 | 145 |
| AMASYA | Boys | 4 | 0 | 4 | 16 | 4 | 0 | 4 | KAYSERİ | Boys | 3 | 1 | 3 | 4 | 2 | 5 | 1 |
|  | Girls | 23 | 33 | 52 | 102 | 119 | 81 | 95 |  | Girls | 57 | 60 | 80 | 146 | 112 | 122 | 159 |
| ANKARA | Boys | 9 | 7 | 6 | 11 | 14 | 11 | 16 | KIRIKKALE | Boys | 0 | 0 | 0 | 5 | 5 | 0 | 10 |
|  | Girls | 141 | 163 | 175 | 305 | 297 | 322 | 289 |  | Girls | 48 | 44 | 89 | 151 | 202 | 147 | 109 |
| ANTALYA | Boys | 2 | 3 | 2 | 4 | 6 | 8 | 7 | KIRKLARELİ | Boys | 0 | 9 | 0 | 0 | 9 | 0 | 0 |
|  | Girls | 66 | 61 | 54 | 109 | 162 | 128 | 134 |  | Girls | 102 | 92 | 65 | 198 | 111 | 155 | 178 |
| ARDAHAN | Boys | 0 | 0 | 0 | 0 | 0 | 0 | 0 | KIRŞEHİR | Boys | 5 | 0 | 10 | 5 | 5 | 5 | 0 |
|  | Girls | 27 | 0 | 14 | 15 | 32 | 0 | 68 |  | Girls | 122 | 135 | 131 | 177 | 198 | 182 | 215 |
| ARTVİN | Boys | 9 | 0 | 0 | 0 | 9 | 9 | 0 | KİLİS | Boys | 6 | 0 | 6 | 6 | 0 | 0 | 0 |
|  | Girls | 39 | 10 | 10 | 51 | 75 | 97 | 68 |  | Girls | 34 | 48 | 95 | 88 | 94 | 79 | 126 |
| AYDIN | Boys | 3 | 0 | 1 | 2 | 2 | 4 | 1 | KOCAELİ | Boys | 1 | 1 | 1 | 2 | 5 | 2 | 6 |
|  | Girls | 25 | 20 | 24 | 95 | 78 | 57 | 41 |  | Girls | 51 | 44 | 49 | 91 | 95 | 101 | 107 |
| BALIKESİR | Boys | 1 | 1 | 1 | 2 | 4 | 2 | 4 | KONYA | Boys | 0 | 1 | 2 | 2 | 3 | 3 | 2 |
|  | Girls | 56 | 29 | 63 | 141 | 134 | 134 | 124 |  | Girls | 48 | 26 | 29 | 89 | 63 | 76 | 72 |
| BARTIN | Boys | 0 | 0 | 23 | 8 | 8 | 24 | 0 | KÜTAHYA | Boys | 2 | 2 | 5 | 3 | 3 | 0 | 11 |
|  | Girls | 94 | 132 | 44 | 255 | 158 | 177 | 215 |  | Girls | 63 | 67 | 110 | 173 | 93 | 123 | 134 |
| BATMAN | Boys | 0 | 0 | 2 | 0 | 0 | 0 | 1 | MALATYA | Boys | 0 | 3 | 1 | 1 | 4 | 3 | 8 |
|  | Girls | 31 | 32 | 33 | 38 | 34 | 34 | 57 |  | Girls | 75 | 59 | 41 | 26 | 136 | 115 | 138 |
| BAYBURT | Boys | 0 | 0 | 0 | 0 | 0 | 0 | 33 | MANİSA | Boys | 0 | 0 | 3 | 3 | 7 | 4 | 1 |
|  | Girls | 16 | 33 | 0 | 17 | 54 | 54 | 57 |  | Girls | 38 | 37 | 35 | 90 | 75 | 81 | 55 |
| BİLECİK | Boys | 0 | 0 | 0 | 0 | 0 | 0 | 6 | MARDİN | Boys | 0 | 2 | 0 | 0 | 3 | 1 | 0 |
|  | Girls | 47 | 47 | 20 | 175 | 135 | 115 | 167 |  | Girls | 14 | 17 | 19 | 24 | 31 | 49 | 43 |
| BİNGÖL | Boys | 0 | 0 | 0 | 0 | 0 | 3 | 0 | MERSİN | Boys | 6 | 3 | 2 | 4 | 3 | 8 | 4 |
|  | Girls | 22 | 41 | 26 | 53 | 38 | 55 | 106 |  | Girls | 55 | 59 | 38 | 61 | 102 | 91 | 106 |
| BİTLİS | Boys | 2 | 0 | 0 | 0 | 0 | 0 | 0 | MUĞLA | Boys | 0 | 5 | 0 | 4 | 3 | 1 | 6 |
|  | Girls | 10 | 12 | 20 | 20 | 31 | 31 | 56 |  | Girls | 45 | 43 | 54 | 110 | 104 | 113 | 59 |
| BOLU | Boys | 0 | 4 | 0 | 0 | 9 | 0 | 10 | MUŞ | Boys | 0 | 0 | 0 | 0 | 2 | 2 | 4 |
|  | Girls | 10 | 41 | 36 | 109 | 48 | 113 | 55 |  | Girls | 6 | 14 | 8 | 16 | 32 | 37 | 55 |
| BURDUR | Boys | 0 | 11 | 5 | 0 | 6 | 0 | 0 | NEVŞEHİR | Boys | 0 | 8 | 0 | 0 | 8 | 4 | 0 |
|  | Girls | 75 | 82 | 96 | 206 | 91 | 123 | 113 |  | Girls | 108 | 94 | 132 | 230 | 117 | 183 | 236 |
| BURSA | Boys | 3 | 3 | 2 | 4 | 6 | 1 | 3 | NİĞDE | Boys | 0 | 0 | 0 | 0 | 0 | 3 | 6 |
|  | Girls | 41 | 43 | 56 | 117 | 116 | 94 | 90 |  | Girls | 69 | 94 | 51 | 114 | 99 | 113 | 96 |
| ÇANAKKALE | Boys | 0 | 8 | 0 | 0 | 0 | 3 | 3 | ORDU | Boys | 2 | 3 | 5 | 5 | 7 | 6 | 2 |
|  | Girls | 52 | 26 | 36 | 56 | 122 | 76 | 64 |  | Girls | 17 | 48 | 33 | 63 | 119 | 117 | 110 |
| ÇANKIRI | Boys | 6 | 0 | 0 | 7 | 7 | 0 | 0 | OSMANİYE | Boys | 0 | 5 | 0 | 0 | 4 | 4 | 0 |
|  | Girls | 86 | 73 | 90 | 213 | 110 | 184 | 123 |  | Girls | 34 | 28 | 36 | 69 | 84 | 96 | 72 |
| ÇORUM | Boys | 5 | 5 | 0 | 5 | 10 | 13 | 9 | RİZE | Boys | 8 | 4 | 4 | 0 | 8 | 0 | 4 |
|  | Girls | 37 | 70 | 54 | 136 | 192 | 85 | 142 |  | Girls | 58 | 23 | 59 | 101 | 94 | 128 | 74 |
| DENİZLİ | Boys | 5 | 6 | 2 | 13 | 9 | 6 | 6 | SAKARYA | Boys | 1 | 2 | 2 | 1 | 0 | 7 | 1 |
|  | Girls | 80 | 98 | 109 | 161 | 119 | 120 | 142 |  | Girls | 34 | 36 | 42 | 96 | 72 | 65 | 70 |
| DİYARBAKIR | Boys | 1 | 2 | 1 | 0 | 1 | 2 | 2 | SAMSUN | Boys | 2 | 2 | 1 | 7 | 6 | 9 | 3 |
|  | Girls | 42 | 63 | 54 | 85 | 95 | 91 | 122 |  | Girls | 38 | 50 | 36 | 154 | 103 | 112 | 101 |
| DÜZCE | Boys | 0 | 0 | 0 | 9 | 6 | 3 | 3 | SİİRT | Boys | 0 | 0 | 0 | 0 | 0 | 0 | 0 |
|  | Girls | 14 | 10 | 3 | 56 | 42 | 46 | 32 |  | Girls | 7 | 20 | 22 | 48 | 41 | 18 | 52 |
| EDİRNE | Boys | 20 | 4 | 0 | 16 | 0 | 12 | 8 | SİNOP | Boys | 7 | 7 | 7 | 7 | 0 | 0 | 0 |
|  | Girls | 73 | 37 | 98 | 154 | 112 | 132 | 145 |  | Girls | 39 | 31 | 40 | 81 | 33 | 49 | 26 |
| ELAZIĞ | Boys | 2 | 2 | 0 | 2 | 4 | 0 | 0 | SİVAS | Boys | 4 | 2 | 0 | 2 | 8 | 4 | 0 |
|  | Girls | 78 | 61 | 56 | 55 | 57 | 125 | 95 |  | Girls | 34 | 57 | 20 | 89 | 245 | 175 | 210 |
| ERZİNCAN | Boys | 0 | 0 | 0 | 5 | 0 | 6 | 0 | ŞANLIURFA | Boys | 1 | 1 | 1 | 1 | 1 | 1 | 1 |
|  | Girls | 18 | 18 | 18 | 81 | 89 | 52 | 87 |  | Girls | 16 | 17 | 35 | 36 | 44 | 36 | 31 |
| ERZURUM | Boys | 1 | 0 | 0 | 0 | 0 | 3 | 1 | ŞIRNAK | Boys | 1 | 0 | 1 | 3 | 1 | 3 | 0 |
|  | Girls | 20 | 16 | 13 | 19 | 33 | 21 | 45 |  | Girls | 13 | 13 | 19 | 27 | 16 | 43 | 38 |
| ESKİŞEHİR | Boys | 2 | 2 | 0 | 8 | 8 | 8 | 13 | TEKİRDAĞ | Boys | 1 | 4 | 2 | 5 | 5 | 4 | 3 |
|  | Girls | 147 | 105 | 72 | 274 | 258 | 249 | 240 |  | Girls | 35 | 51 | 46 | 129 | 93 | 85 | 85 |
| GAZİANTEP | Boys | 0 | 2 | 1 | 3 | 4 | 2 | 3 | TOKAT | Boys | 0 | 0 | 0 | 0 | 2 | 5 | 0 |
|  | Girls | 26 | 42 | 61 | 91 | 87 | 92 | 109 |  | Girls | 53 | 72 | 43 | 106 | 118 | 150 | 194 |
| GİRESUN | Boys | 3 | 10 | 0 | 0 | 0 | 4 | 4 | TRABZON | Boys | 6 | 14 | 6 | 9 | 13 | 10 | 12 |
|  | Girls | 39 | 59 | 52 | 53 | 116 | 128 | 151 |  | Girls | 116 | 69 | 88 | 136 | 153 | 212 | 190 |
| GÜMÜŞHANE | Boys | 0 | 8 | 0 | 10 | 10 | 0 | 0 | TUNCELİ | Boys | 0 | 0 | 0 | 0 | 0 | 0 | 0 |
|  | Girls | 29 | 10 | 44 | 76 | 35 | 94 | 63 |  | Girls | 43 | 43 | 22 | 108 | 22 | 43 | 45 |
| HAKKARİ | Boys | 0 | 0 | 0 | 0 | 3 | 0 | 0 | UŞAK | Boys | 0 | 0 | 0 | 0 | 4 | 0 | 12 |
|  | Girls | 3 | 4 | 18 | 36 | 22 | 15 | 23 |  | Girls | 69 | 103 | 96 | 157 | 94 | 114 | 109 |
| HATAY | Boys | 1 | 1 | 0 | 1 | 1 | 2 | 2 | VAN | Boys | 1 | 1 | 0 | 1 | 2 | 1 | 3 |
|  | Girls | 26 | 24 | 22 | 49 | 53 | 61 | 76 |  | Girls | 7 | 12 | 35 | 35 | 47 | 50 | 49 |
| IĞDIR | Boys | 0 | 0 | 0 | 4 | 0 | 0 | 0 | YALOVA | Boys | 5 | 9 | 13 | 4 | 0 | 9 | 9 |
|  | Girls | 5 | 14 | 28 | 48 | 59 | 30 | 63 |  | Girls | 32 | 62 | 57 | 147 | 113 | 83 | 94 |
| ISPARTA | Boys | 0 | 0 | 3 | 0 | 0 | 3 | 0 | YOZGAT | Boys | 0 | 6 | 0 | 0 | 6 | 3 | 0 |
|  | Girls | 84 | 42 | 96 | 122 | 149 | 89 | 65 |  | Girls | 77 | 73 | 55 | 96 | 77 | 128 | 321 |
| İSTANBUL | Boys | 4 | 4 | 3 | 3 | 5 | 4 | 5 | ZONGULDAK | Boys | 2 | 0 | 5 | 5 | 3 | 6 | 9 |
|  | Girls | 57 | 58 | 66 | 131 | 125 | 120 | 123 |  | Girls | 37 | 58 | 55 | 178 | 188 | 117 | 201 |
| İZMİR | Boys | 4 | 1 | 3 | 5 | 6 | 7 | 7 |  | | | | | | | | |
|  | Girls | 43 | 45 | 52 | 111 | 107 | 96 | 90 |  |  |  |  |  |  |  |  |  |

Incidence was calculated per 100,000 population using individuals aged 11 and under for boys, and 10 and under for girls.
