# Supplementary material for: Central precocious puberty in Türkiye, 2018–2024: national incidence, prevalence, and changes across the COVID-19 period
Source: Front Endocrinol (Lausanne). 2026 Mar 10;17:1773426. doi: 10.3389/fendo.2026.1773426 (PMC13008741; doi:10.3389/fendo.2026.1773426)
Supplement: Supplementary file 2 [file Table2.docx]

Supplementary Table 2. Prevalence by Province and Sex (per 100,000 People)

|  |  | 2018 | 2019 | 2020 | 2021 | 2022 | 2023 | 2024 |  |  | 2018 | 2019 | 2020 | 2021 | 2022 | 2023 | 2024 |  |  | 2018 | 2019 | 2020 | 2021 | 2022 | 2023 | 2024 |
| --- | --- | --- | --- | --- | --- | --- | --- | --- | --- | --- | --- | --- | --- | --- | --- | --- | --- | --- | --- | --- | --- | --- | --- | --- | --- | --- |
| ADANA | Boys | 1 | 2 | 4 | 7 | 9 | 9 | 5 | EDİRNE | Boys | 18 | 18 | 11 | 19 | 15 | 15 | 15 | MALATYA | Boys | 0 | 2 | 4 | 1 | 5 | 7 | 12 |
|  | Girls | 33 | 66 | 88 | 115 | 163 | 160 | 155 |  | Girls | 67 | 89 | 154 | 228 | 304 | 300 | 302 |  | Girls | 68 | 98 | 100 | 42 | 173 | 230 | 262 |
|  | Total | 16 | 32 | 43 | 57 | 81 | 80 | 75 |  | Total | 41 | 51 | 77 | 115 | 149 | 147 | 148 |  | Total | 32 | 47 | 49 | 20 | 83 | 111 | 128 |
| ADIYAMAN | Boys | 2 | 1 | 1 | 1 | 1 | 1 | 1 | ELAZIĞ | Boys | 2 | 3 | 2 | 2 | 5 | 4 | 4 | MANİSA | Boys | 0 | 0 | 2 | 5 | 10 | 12 | 9 |
|  | Girls | 28 | 35 | 40 | 50 | 111 | 112 | 123 |  | Girls | 71 | 120 | 124 | 130 | 142 | 214 | 214 |  | Girls | 35 | 64 | 74 | 124 | 161 | 167 | 154 |
|  | Total | 14 | 17 | 19 | 24 | 53 | 53 | 58 |  | Total | 34 | 58 | 58 | 61 | 69 | 101 | 101 |  | Total | 16 | 30 | 36 | 60 | 80 | 84 | 76 |
| AFYONKARAHİSAR | Boys | 0 | 0 | 1 | 3 | 7 | 4 | 15 | ERZİNCAN | Boys | 0 | 0 | 0 | 5 | 5 | 5 | 5 | MARDİN | Boys | 0 | 2 | 2 | 1 | 4 | 4 | 3 |
|  | Girls | 30 | 76 | 104 | 182 | 201 | 213 | 218 |  | Girls | 17 | 34 | 34 | 91 | 156 | 147 | 152 |  | Girls | 13 | 29 | 42 | 45 | 57 | 81 | 100 |
|  | Total | 14 | 35 | 49 | 86 | 97 | 101 | 109 |  | Total | 8 | 16 | 16 | 45 | 76 | 71 | 73 |  | Total | 6 | 14 | 21 | 21 | 29 | 40 | 49 |
| AĞRI | Boys | 0 | 0 | 0 | 1 | 4 | 6 | 3 | ERZURUM | Boys | 1 | 1 | 1 | 1 | 0 | 3 | 4 | MERSİN | Boys | 5 | 6 | 5 | 6 | 9 | 12 | 14 |
|  | Girls | 4 | 7 | 10 | 23 | 47 | 58 | 51 |  | Girls | 18 | 29 | 31 | 33 | 55 | 48 | 70 |  | Girls | 50 | 85 | 73 | 83 | 169 | 186 | 201 |
|  | Total | 2 | 3 | 4 | 11 | 24 | 30 | 26 |  | Total | 9 | 14 | 15 | 16 | 26 | 24 | 35 |  | Total | 26 | 43 | 37 | 42 | 83 | 93 | 101 |
| AKSARAY | Boys | 2 | 2 | 2 | 2 | 9 | 7 | 5 | ESKİŞEHİR | Boys | 1 | 3 | 3 | 7 | 11 | 16 | 19 | MUĞLA | Boys | 0 | 5 | 4 | 4 | 6 | 8 | 10 |
|  | Girls | 47 | 90 | 112 | 223 | 315 | 314 | 271 |  | Girls | 134 | 210 | 175 | 305 | 462 | 438 | 363 |  | Girls | 41 | 70 | 96 | 165 | 222 | 231 | 193 |
|  | Total | 23 | 43 | 54 | 106 | 152 | 151 | 129 |  | Total | 63 | 99 | 82 | 145 | 220 | 211 | 178 |  | Total | 19 | 35 | 47 | 78 | 107 | 111 | 95 |
| AMASYA | Boys | 4 | 4 | 4 | 19 | 8 | 8 | 8 | GAZİANTEP | Boys | 0 | 2 | 2 | 4 | 7 | 7 | 8 | MUŞ | Boys | 0 | 0 | 0 | 0 | 2 | 4 | 6 |
|  | Girls | 21 | 38 | 73 | 136 | 239 | 233 | 248 |  | Girls | 24 | 57 | 96 | 140 | 184 | 192 | 204 |  | Girls | 5 | 18 | 20 | 27 | 53 | 72 | 105 |
|  | Total | 12 | 20 | 36 | 73 | 114 | 111 | 118 |  | Total | 12 | 28 | 46 | 68 | 90 | 94 | 100 |  | Total | 3 | 8 | 9 | 13 | 26 | 36 | 53 |
| ANKARA | Boys | 8 | 13 | 13 | 16 | 24 | 28 | 31 | GİRESUN | Boys | 3 | 12 | 13 | 0 | 0 | 3 | 7 | NEVŞEHİR | Boys | 0 | 7 | 7 | 0 | 7 | 7 | 4 |
|  | Girls | 129 | 243 | 295 | 421 | 589 | 613 | 591 |  | Girls | 35 | 82 | 94 | 88 | 181 | 248 | 288 |  | Girls | 98 | 164 | 220 | 341 | 346 | 337 | 368 |
|  | Total | 65 | 120 | 144 | 204 | 287 | 301 | 291 |  | Total | 18 | 45 | 50 | 41 | 84 | 117 | 137 |  | Total | 46 | 80 | 106 | 157 | 164 | 160 | 172 |
| ANTALYA | Boys | 2 | 4 | 5 | 6 | 11 | 14 | 17 | GÜMÜŞHANE | Boys | 0 | 8 | 9 | 18 | 19 | 19 | 10 | NİĞDE | Boys | 0 | 0 | 0 | 0 | 0 | 3 | 9 |
|  | Girls | 61 | 104 | 118 | 151 | 272 | 294 | 295 |  | Girls | 26 | 26 | 51 | 110 | 118 | 173 | 181 |  | Girls | 63 | 140 | 146 | 161 | 203 | 205 | 204 |
|  | Total | 29 | 51 | 57 | 73 | 132 | 143 | 146 |  | Total | 12 | 16 | 29 | 61 | 66 | 91 | 91 |  | Total | 29 | 66 | 68 | 75 | 95 | 97 | 99 |
| ARDAHAN | Boys | 0 | 0 | 0 | 0 | 0 | 0 | 0 | HAKKARİ | Boys | 0 | 0 | 0 | 0 | 3 | 0 | 3 | ORDU | Boys | 2 | 5 | 8 | 8 | 17 | 19 | 14 |
|  | Girls | 25 | 13 | 26 | 41 | 43 | 30 | 62 |  | Girls | 3 | 6 | 23 | 46 | 54 | 51 | 42 |  | Girls | 16 | 57 | 59 | 89 | 182 | 231 | 233 |
|  | Total | 12 | 6 | 12 | 19 | 20 | 14 | 29 |  | Total | 1 | 3 | 11 | 22 | 27 | 24 | 21 |  | Total | 8 | 29 | 32 | 46 | 93 | 117 | 115 |
| ARTVİN | Boys | 8 | 8 | 0 | 0 | 9 | 9 | 9 | HATAY | Boys | 0 | 1 | 1 | 1 | 3 | 3 | 4 | OSMANİYE | Boys | 0 | 5 | 3 | 2 | 3 | 7 | 3 |
|  | Girls | 36 | 37 | 46 | 75 | 117 | 177 | 204 |  | Girls | 24 | 42 | 53 | 67 | 111 | 137 | 151 |  | Girls | 31 | 53 | 63 | 98 | 158 | 186 | 171 |
|  | Total | 21 | 22 | 22 | 35 | 59 | 87 | 100 |  | Total | 12 | 21 | 25 | 32 | 53 | 65 | 72 |  | Total | 14 | 27 | 31 | 47 | 75 | 90 | 81 |
| AYDIN | Boys | 3 | 3 | 2 | 4 | 3 | 7 | 8 | IĞDIR | Boys | 0 | 0 | 0 | 4 | 4 | 0 | 0 | RİZE | Boys | 7 | 11 | 7 | 7 | 11 | 11 | 8 |
|  | Girls | 23 | 39 | 43 | 111 | 161 | 146 | 111 |  | Girls | 4 | 17 | 43 | 65 | 117 | 101 | 125 |  | Girls | 53 | 66 | 112 | 143 | 214 | 259 | 232 |
|  | Total | 13 | 20 | 21 | 54 | 77 | 72 | 56 |  | Total | 2 | 8 | 20 | 33 | 57 | 47 | 58 |  | Total | 28 | 37 | 56 | 70 | 106 | 127 | 112 |
| BALIKESİR | Boys | 1 | 2 | 3 | 2 | 7 | 9 | 9 | ISPARTA | Boys | 0 | 0 | 3 | 3 | 0 | 3 | 3 | SAKARYA | Boys | 1 | 3 | 3 | 2 | 3 | 8 | 6 |
|  | Girls | 51 | 69 | 104 | 180 | 261 | 282 | 255 |  | Girls | 76 | 77 | 133 | 190 | 269 | 201 | 136 |  | Girls | 31 | 59 | 81 | 133 | 181 | 174 | 162 |
|  | Total | 24 | 33 | 50 | 85 | 125 | 136 | 123 |  | Total | 35 | 36 | 63 | 90 | 125 | 95 | 65 |  | Total | 15 | 29 | 40 | 63 | 86 | 86 | 79 |
| BARTIN | Boys | 0 | 0 | 21 | 14 | 29 | 44 | 38 | İSTANBUL | Boys | 4 | 7 | 7 | 6 | 10 | 10 | 11 | SAMSUN | Boys | 2 | 3 | 4 | 9 | 14 | 19 | 16 |
|  | Girls | 85 | 183 | 121 | 313 | 363 | 425 | 466 |  | Girls | 52 | 95 | 118 | 183 | 264 | 278 | 273 |  | Girls | 35 | 76 | 86 | 188 | 260 | 255 | 244 |
|  | Total | 40 | 85 | 67 | 153 | 183 | 220 | 236 |  | Total | 26 | 48 | 59 | 88 | 128 | 135 | 133 |  | Total | 17 | 37 | 43 | 92 | 128 | 129 | 122 |
| BATMAN | Boys | 0 | 0 | 2 | 2 | 1 | 0 | 1 | İZMİR | Boys | 3 | 3 | 5 | 8 | 12 | 15 | 16 | SİİRT | Boys | 0 | 0 | 0 | 0 | 0 | 0 | 0 |
|  | Girls | 28 | 54 | 66 | 65 | 86 | 82 | 105 |  | Girls | 40 | 72 | 98 | 145 | 229 | 227 | 212 |  | Girls | 7 | 23 | 39 | 63 | 95 | 84 | 92 |
|  | Total | 13 | 26 | 32 | 32 | 41 | 39 | 50 |  | Total | 20 | 35 | 49 | 72 | 113 | 114 | 107 |  | Total | 3 | 11 | 18 | 30 | 45 | 39 | 43 |
| BAYBURT | Boys | 0 | 0 | 0 | 0 | 0 | 0 | 31 | KAHRAMANMARAŞ | Boys | 3 | 5 | 4 | 4 | 4 | 3 | 2 | SİNOP | Boys | 6 | 12 | 13 | 13 | 13 | 13 | 0 |
|  | Girls | 15 | 30 | 15 | 16 | 66 | 98 | 120 |  | Girls | 44 | 82 | 106 | 132 | 165 | 155 | 161 |  | Girls | 35 | 64 | 65 | 126 | 152 | 141 | 100 |
|  | Total | 7 | 14 | 7 | 7 | 31 | 46 | 73 |  | Total | 22 | 41 | 51 | 63 | 79 | 74 | 76 |  | Total | 20 | 37 | 37 | 66 | 78 | 73 | 47 |
| BİLECİK | Boys | 0 | 0 | 0 | 0 | 0 | 0 | 6 | KARABÜK | Boys | 0 | 6 | 6 | 6 | 18 | 18 | 12 | SİVAS | Boys | 3 | 5 | 4 | 2 | 9 | 13 | 8 |
|  | Girls | 43 | 73 | 61 | 173 | 247 | 228 | 220 |  | Girls | 96 | 150 | 185 | 221 | 293 | 289 | 321 |  | Girls | 31 | 77 | 67 | 93 | 306 | 328 | 385 |
|  | Total | 20 | 35 | 29 | 81 | 116 | 108 | 107 |  | Total | 44 | 72 | 89 | 106 | 146 | 143 | 155 |  | Total | 16 | 39 | 33 | 44 | 148 | 159 | 183 |
| BİNGÖL | Boys | 0 | 0 | 0 | 0 | 0 | 3 | 3 | KARAMAN | Boys | 0 | 4 | 8 | 4 | 8 | 0 | 0 | ŞANLIURFA | Boys | 1 | 2 | 2 | 1 | 2 | 3 | 3 |
|  | Girls | 20 | 54 | 58 | 76 | 103 | 119 | 183 |  | Girls | 5 | 19 | 23 | 61 | 120 | 168 | 168 |  | Girls | 15 | 29 | 55 | 61 | 96 | 94 | 83 |
|  | Total | 9 | 25 | 27 | 36 | 48 | 57 | 87 |  | Total | 2 | 11 | 15 | 31 | 60 | 78 | 79 |  | Total | 7 | 15 | 27 | 29 | 46 | 46 | 41 |
| BİTLİS | Boys | 2 | 0 | 0 | 0 | 0 | 0 | 0 | KARS | Boys | 0 | 0 | 0 | 0 | 3 | 3 | 11 | ŞIRNAK | Boys | 1 | 1 | 2 | 3 | 4 | 7 | 6 |
|  | Girls | 9 | 20 | 34 | 32 | 59 | 66 | 85 |  | Girls | 13 | 17 | 24 | 29 | 72 | 82 | 99 |  | Girls | 12 | 21 | 30 | 38 | 51 | 71 | 91 |
|  | Total | 5 | 9 | 16 | 15 | 27 | 31 | 39 |  | Total | 6 | 8 | 11 | 13 | 35 | 40 | 51 |  | Total | 6 | 11 | 15 | 20 | 26 | 37 | 46 |

|  |  | 2018 | 2019 | 2020 | 2021 | 2022 | 2023 | 2024 |  |  | 2018 | 2019 | 2020 | 2021 | 2022 | 2023 | 2024 |  |  | 2018 | 2019 | 2020 | 2021 | 2022 | 2023 | 2024 |
| --- | --- | --- | --- | --- | --- | --- | --- | --- | --- | --- | --- | --- | --- | --- | --- | --- | --- | --- | --- | --- | --- | --- | --- | --- | --- | --- |
| BOLU | Boys | 0 | 4 | 4 | 0 | 13 | 9 | 9 | KASTAMONU | Boys | 0 | 0 | 0 | 15 | 24 | 28 | 24 | TEKİRDAĞ | Boys | 1 | 5 | 7 | 7 | 10 | 10 | 10 |
|  | Girls | 9 | 42 | 51 | 128 | 144 | 200 | 160 |  | Girls | 76 | 146 | 144 | 170 | 263 | 287 | 287 |  | Girls | 32 | 76 | 85 | 158 | 229 | 205 | 195 |
|  | Total | 4 | 21 | 26 | 59 | 74 | 98 | 79 |  | Total | 35 | 68 | 67 | 87 | 135 | 148 | 146 |  | Total | 15 | 38 | 43 | 77 | 113 | 101 | 96 |
| BURDUR | Boys | 0 | 10 | 10 | 0 | 10 | 5 | 0 | KAYSERİ | Boys | 3 | 3 | 5 | 6 | 7 | 9 | 8 | TOKAT | Boys | 0 | 0 | 0 | 0 | 2 | 6 | 6 |
|  | Girls | 68 | 139 | 163 | 258 | 243 | 242 | 230 |  | Girls | 52 | 92 | 129 | 201 | 254 | 250 | 268 |  | Girls | 48 | 103 | 99 | 136 | 234 | 269 | 336 |
|  | Total | 31 | 70 | 81 | 120 | 118 | 115 | 107 |  | Total | 26 | 44 | 62 | 97 | 122 | 121 | 129 |  | Total | 22 | 48 | 46 | 63 | 109 | 128 | 159 |
| BURSA | Boys | 3 | 4 | 4 | 6 | 11 | 8 | 5 | KIRIKKALE | Boys | 0 | 0 | 0 | 5 | 9 | 5 | 10 | TRABZON | Boys | 6 | 19 | 21 | 25 | 32 | 34 | 32 |
|  | Girls | 37 | 66 | 90 | 149 | 215 | 204 | 172 |  | Girls | 43 | 74 | 117 | 190 | 342 | 336 | 293 |  | Girls | 106 | 165 | 184 | 229 | 332 | 411 | 437 |
|  | Total | 19 | 33 | 44 | 72 | 106 | 99 | 82 |  | Total | 20 | 34 | 54 | 90 | 163 | 158 | 141 |  | Total | 53 | 87 | 98 | 120 | 172 | 210 | 221 |
| ÇANAKKALE | Boys | 0 | 8 | 5 | 5 | 0 | 3 | 3 | KIRKLARELİ | Boys | 0 | 8 | 8 | 4 | 8 | 4 | 0 | TUNCELİ | Boys | 0 | 0 | 0 | 0 | 0 | 0 | 0 |
|  | Girls | 48 | 63 | 60 | 69 | 180 | 201 | 125 |  | Girls | 93 | 158 | 171 | 264 | 311 | 318 | 348 |  | Girls | 40 | 60 | 40 | 121 | 143 | 99 | 125 |
|  | Total | 22 | 33 | 31 | 35 | 84 | 95 | 60 |  | Total | 43 | 78 | 84 | 125 | 147 | 149 | 161 |  | Total | 19 | 28 | 19 | 56 | 67 | 46 | 58 |
| ÇANKIRI | Boys | 6 | 6 | 7 | 7 | 13 | 7 | 0 | KIRŞEHİR | Boys | 5 | 5 | 14 | 10 | 15 | 15 | 5 | UŞAK | Boys | 0 | 0 | 0 | 0 | 3 | 3 | 14 |
|  | Girls | 79 | 133 | 173 | 263 | 295 | 282 | 288 |  | Girls | 111 | 203 | 228 | 301 | 348 | 361 | 366 |  | Girls | 63 | 151 | 191 | 233 | 251 | 254 | 264 |
|  | Total | 40 | 66 | 84 | 126 | 144 | 135 | 134 |  | Total | 55 | 98 | 114 | 146 | 171 | 177 | 173 |  | Total | 29 | 69 | 88 | 107 | 117 | 119 | 129 |
| ÇORUM | Boys | 4 | 9 | 9 | 7 | 17 | 20 | 26 | KİLİS | Boys | 5 | 5 | 11 | 11 | 11 | 0 | 0 | VAN | Boys | 1 | 1 | 1 | 1 | 3 | 4 | 7 |
|  | Girls | 34 | 94 | 107 | 189 | 322 | 283 | 293 |  | Girls | 31 | 75 | 151 | 168 | 218 | 187 | 201 |  | Girls | 7 | 18 | 44 | 60 | 93 | 106 | 110 |
|  | Total | 18 | 48 | 54 | 91 | 158 | 142 | 149 |  | Total | 17 | 38 | 76 | 85 | 107 | 88 | 94 |  | Total | 3 | 9 | 22 | 29 | 45 | 52 | 55 |
| DENİZLİ | Boys | 4 | 9 | 8 | 19 | 24 | 26 | 22 | KOCAELİ | Boys | 1 | 2 | 1 | 3 | 7 | 7 | 10 | YALOVA | Boys | 4 | 12 | 25 | 16 | 12 | 16 | 20 |
|  | Girls | 73 | 147 | 181 | 254 | 285 | 278 | 269 |  | Girls | 47 | 81 | 89 | 123 | 194 | 205 | 222 |  | Girls | 29 | 75 | 108 | 167 | 229 | 209 | 171 |
|  | Total | 36 | 73 | 88 | 129 | 146 | 144 | 137 |  | Total | 23 | 39 | 42 | 59 | 94 | 100 | 109 |  | Total | 16 | 42 | 64 | 86 | 114 | 106 | 90 |
| DİYARBAKIR | Boys | 1 | 3 | 3 | 2 | 3 | 4 | 6 | KONYA | Boys | 0 | 1 | 3 | 4 | 6 | 8 | 7 | YOZGAT | Boys | 0 | 5 | 3 | 3 | 9 | 9 | 6 |
|  | Girls | 39 | 92 | 121 | 141 | 196 | 219 | 250 |  | Girls | 44 | 60 | 60 | 111 | 145 | 150 | 147 |  | Girls | 70 | 130 | 134 | 158 | 167 | 222 | 395 |
|  | Total | 19 | 45 | 58 | 67 | 93 | 104 | 120 |  | Total | 21 | 29 | 29 | 54 | 71 | 74 | 72 |  | Total | 32 | 63 | 64 | 75 | 82 | 108 | 187 |
| DÜZCE | Boys | 0 | 0 | 0 | 8 | 14 | 8 | 9 | KÜTAHYA | Boys | 2 | 4 | 9 | 7 | 10 | 7 | 13 | ZONGULDAK | Boys | 2 | 2 | 7 | 9 | 10 | 10 | 13 |
|  | Girls | 13 | 16 | 13 | 54 | 90 | 110 | 72 |  | Girls | 57 | 94 | 175 | 272 | 290 | 293 | 275 |  | Girls | 34 | 78 | 84 | 206 | 337 | 288 | 357 |
|  | Total | 6 | 7 | 6 | 30 | 50 | 56 | 38 |  | Total | 28 | 47 | 87 | 131 | 140 | 141 | 135 |  | Total | 17 | 37 | 42 | 101 | 161 | 139 | 173 |

Prevalence was calculated per 100,000 population using individuals aged 12 and under for boys, and 11 and under for girls.
